# Acute *Plasmodium yoelii* 17XNL Infection During BCG Vaccination Limits T Cell Responses and Mycobacterial Growth Inhibition

**DOI:** 10.1111/imm.70006

**Published:** 2025-06-19

**Authors:** Emily Tangie, Nawamin Pinpathomrat, Rachel Tanner, Nai‐Jen Hsu, Alexandra J. Spencer, Muazzam Jacobs, Helen Mcshane, Roanne Keeton

**Affiliations:** ^1^ Division of Immunology, Department of Pathology, Faculty of Health Sciences University of Cape Town Cape Town South Africa; ^2^ Jenner Institute, Nuffield Department of Medicine University of Oxford Oxford UK; ^3^ Department of Biomedical Sciences and Biomedical Engineering, Faculty of Medicine Prince of Songkla University Songkhla Thailand; ^4^ Department of Biology University of Oxford Oxford UK; ^5^ School of Biomedical Sciences and Pharmacy University of Newcastle Newcastle Australia; ^6^ National Health Laboratory Service Cape Town South Africa; ^7^ Institute of Infectious Disease and Molecular Medicine, Faculty of Health Sciences University of Cape Town Cape Town South Africa; ^8^ Neuroscience Institute, Faculty of Health Sciences University of Cape Town Cape Town South Africa

**Keywords:** BCG, cytokines, malaria, mycobacterial growth inhibition assay, tuberculosis

## Abstract

Tuberculosis and malaria overlap in many sub‐Saharan African countries where Bacillus Calmette Guérin (BCG) vaccination is routinely administered. The aim of this study was to determine whether the timing of BCG vaccination in relation to a malaria infection has implications for BCG vaccine efficacy. Mice were intradermally vaccinated with BCG either 4 weeks before infection with blood‐stage *Plasmodium yoelii 17XNL*, at 13 days post‐infection (during an acute blood‐stage malaria infection) or 21 days post‐infection (after clearance of *P. yoelii 17XNL* infection). Ex vivo control of mycobacterial growth by splenocytes was used as a surrogate of protective efficacy, and PPD‐specific T‐cell responses were quantified by flow cytometry. No differences in mycobacterial growth control were detected between BCG vaccinated mice and groups receiving vaccination prior to or after clearance of *P. yoelii 17XNL* infection. Poorer control of mycobacterial growth was observed following BCG vaccination administered during an acute malarial infection compared to BCG vaccination only or BCG vaccination after blood‐stage malaria infection, and mycobacterial growth negatively correlated with the magnitude of total cytokine production from PPD‐specific CD4^+^ T cells (*p* < 0.0001). Delayed BCG vaccination beyond the neonatal period may increase the risk of concurrent malarial infections with the potential to reduce BCG efficacy in children in malaria‐endemic areas.

## Graphical abstract

Tuberculosis and malaria overlap in many sub‐Saharan African countries where BCG vaccination is routinely administered. The aim of this study was to determine whether the timing of BCG vaccination in relation to a malaria infection has implications for BCG vaccine efficacy. We demonstrated poorer control of mycobacterial growth following BCG vaccination administered during an acute malarial infection, which negatively correlated with the magnitude of total cytokine production from PPD‐specific CD4^+^ T cells.

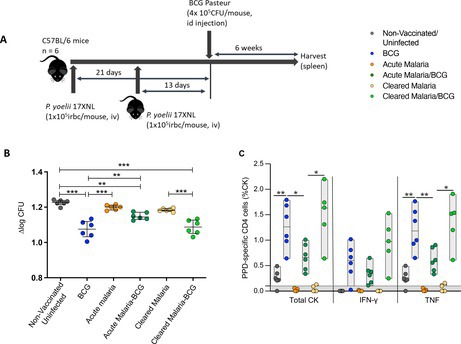

## 1 Introduction

Tuberculosis (TB) remains a global public health concern, with an estimated 2 billion people—just under a quarter of the world's population—infected with *Mycobacterium tuberculosis* (*M. tb*) [1, 2]. Amongst those infected, about 10% progress to active TB disease during their lifetime [3]. Although the annual number of deaths from TB was reported to be decreasing globally, with an estimated cumulative reduction of 14% between 2015 and 2019 [2], this trend was reversed with the onset of the COVID‐19 pandemic and redirection of resources. A global estimate of 10.8 million people fell ill of TB in 2023 [2]. Control measures for TB make use of a standard six‐month drug treatment regimen, but the emergence of multidrug‐resistant and extremely drug‐resistant strains of *M. tb* exacerbates the problem [4, 5, 6]. Control of the TB epidemic is further compounded by the lack of an effective vaccine for the prevention of the disease, and despite a number of vaccine candidates currently in clinical trials, vaccine research and development is difficult given the lack of validated immune correlates of protection.

Even though vaccination remains the most cost‐effective strategy for TB prevention, Bacillus Calmette Guérin (BCG) remains the only vaccine that has been licensed for use against TB since 1921. There are numerous TB vaccine candidates at different phases of clinical trials [7, 8] with amongst the most promising being MTBVAC (an attenuated strain of *M. tb*) and M72/AS01E, an adjuvant fusion protein subunit vaccine [9]. BCG protects against severe disseminated forms of TB in infants and may also confer non‐specific protection against all‐cause mortality [10, 11]. However, BCG offers highly variable levels of protection against pulmonary TB ranging from 0% to 80% [12, 13, 14]. The poor efficacy has been largely attributed to exposure to environmental non‐tuberculous mycobacteria (NTM), although human and mycobacterial genetics, coinfections, and geographical location may also play a role [15, 16, 17].

Malaria, like TB, continues to cause severe morbidity and mortality worldwide despite global efforts to eradicate the disease. In 2023, a global estimate of 263 million malaria cases was reported, with the largest proportions of deaths occurring in children under the age of 5 living in the WHO Africa Region [18]. Previous studies have indicated that malaria induces dysregulation of innate and adaptive anti‐mycobacterial defences [19]. Malaria and TB overlap in many sub‐Saharan countries where BCG is routinely administered [2, 18]. The geographical overlap between these two diseases increases the likelihood of a co‐infection that could, in turn, alter the host's ability to generate specific immune responses against *M. tb*. Studies by Scott, Hawkes, and colleagues have reported that malaria exacerbates mycobacterial disease in acute and latent infection models [19, 20], while others have shown that malaria can skew T cell responses towards a Th2 cytokine type response [21] which may reduce the inflammatory damage caused by severe TB disease or exacerbate disease progression.

It has been suggested that malaria reduces the efficacy of the measles vaccine in infants [22], and it may similarly influence the effectiveness of BCG. The timing of BCG vaccination with respect to malaria exposure might be crucial in determining the outcome. While BCG policies vary amongst different countries, in high burden settings, BCG is usually recommended for administration a few days after birth. However, in malaria‐endemic regions, BCG vaccination can be delayed by factors such as vaccine shortage, health care infrastructure, financial limitations, and logistical challenges, which could increase the likelihood of malaria exposure after birth and prior to BCG administration [23, 24]. It has been demonstrated that delaying BCG vaccination from birth to 10 weeks of age enhances the quantitative and qualitative BCG‐specific T cell response [25]; however, a delay may increase the likelihood of infant malaria infection prior to vaccination in endemic regions. We hypothesise that prior exposure to malaria may reduce the efficacy of BCG vaccination through altering the vaccine‐specific T cell responses. We have previously reported that BCG mediated protection against an in vivo *M. tb* aerosol challenge is sustained post‐malaria infection irrespective of malarial virulence [26]. In the current study, we sought to confirm this in another mouse model, *Plasmodium yoelii* (*P. yoelii 17XNL*), and evaluate whether malaria exposure to low virulence *P. yoelii 17XNL* around the time of vaccination would alter BCG induced protection, using the mycobacterial growth inhibition assay (MGIA) as a surrogate of protective efficacy. The MGIA is increasingly being used as an important tool for the preclinical evaluation of vaccine candidates. Several studies have reported comparable results in BCG‐induced protection using MGIA and after in vivo aerosol challenge [27, 28, 29, 30].

## 2 Materials and Methods

### 2.1 Sex as a Biological Variable

This study exclusively examined female C57BL/6 mice. Although sex differences have been shown as a biological variable in vaccine immunity [31], the focus of our study is to determine any detrimental effects of malarial co‐infection on BCG efficacy. To dissociate these effects from other variables, only female mice were used in the current study.

### 2.2 Mouse Experiments

Wild‐type female C57BL/6OlaHsd (C57BL/6) mice of 6 weeks were acquired from Envigo (UK). Five to six animals were randomly assigned per cage on arrival at the facility. Due to the nature of the studies, with each group having a unique sequence of BCG vaccinations and *P. yoelii 17XNL* infections, blinding was not feasible. The sample size was determined based on published data for similar studies [26, 32]. The mouse distribution, timing of BCG vaccination and *P. yoelii 17XNL* infection, number of mice per group, analysis done, and timing, and any missing data points with reasons for each of Studies 1, 2a, and 2b are outlined in Tables S1–S3.

Animals were group housed in individually ventilated cages under SPF conditions, with constant temperature and humidity, with lighting on a 12:12 (8 am–8 pm) light–dark cycle. To support mouse welfare, they were provided with environmental enrichment including paper for shredding and burrowing, chewable material, and red mouse houses or tunnels for hiding and play. Prior to the start of each study, they were given a minimum of 7 days for acclimatisation. To minimise stress during the study, handling was kept to a minimum and only done by experienced personnel trained in refined animal handling techniques, and any procedures that may cause discomfort were done under short‐term anaesthesia using vaporised IsoFlo. All animals were humanely sacrificed at the end of each experiment (timing shown Tables S1–S3) by cervical dislocation following standard approaches to minimise distress in accordance with the Oxford University Gold Standard Policy, which included only having one cage of mice present in the procedure room at a time.

### 2.3 BCG Immunisation

For the immunisation, mid‐log phase BCG Pasteur frozen stocks previously cultured in‐house in 7H9 broth (BD, UK) enriched with 10% Middlebrook OADC and 0.05% Tween 80 were used. The stocks were thawed at room temperature and sonicated for 1 min to dissociate clumps. The sonicated BCG was then diluted to 8 × 10^6^ CFU per ml in PBS. Each animal was vaccinated with 50 μL of the 8 × 10^6^ CFU per ml BCG stock to achieve a final concentration of 4 × 10^5^ CFU per mouse. This was administered as 25 μL intradermal injections into each of the left and right ear lobes. The timing of BCG vaccination for each study is detailed in Tables S1–S3.

### 2.4 Plasmodium Parasite Infection

In this study, we will refer to *Plasmodium yoelii 17XNL* as *P. yoelii 17XNL* for simplicity. To prepare a viable preparation of *P. yoelii 17XNL* infected red blood cells (RBCs) for inoculation of experimental mice, frozen blood stocks, containing > 1% *P. yoelii 17XNL* infected RBCs, were passaged through a donor mouse. Briefly, frozen blood stocks of 1% *P. yoelii 17XNL* were thawed, diluted to 1 × 10^6^ infected RBCs per ml, and 100 μL injected intravenously into the tail vein of donor mice. Parasitaemia was monitored daily by Giemsa staining of thin blood films. When parasitaemia in the donor mouse reached greater than 1%, the mouse was sacrificed, blood collected, and diluted in PBS to a concentration of 1 × 10^6^ *P. yoelii 17XNL* infected RBCs per ml such that a 100 μL tail vein intravenous injection would deliver the required infection dose of 1 × 10^5^ *P. yoelii 17XNL* infected RBCs into each experimental mouse. The timing of *P. yoelii 17XNL* infection for each study is detailed in Tables S1–S3.

### 2.5 Flow Cytometry

#### 2.5.1 Splenocyte Harvesting and Preparation

All C57BL/6 mice were euthanized at the end of the experiments. Spleens were harvested post‐mortem by making an incision on the left ventral side of the mouse, resecting the peritoneum and removing the spleen aseptically from the surrounding connective tissue. Individual spleens were processed in PBS through a 70 μm cell strainer (Falcon 352 350). The RBCs were lysed in 5 mL of ammonium‐chloride‐potassium lysing buffer (ACK buffer) for 5 min. After washing, the cells were resuspended in 10% FCS in RPMI medium without antibiotics and counted using a Casy counter (Roche, Basel, Switzerland). The cells were further diluted in RPMI medium to achieve the desired number of cells for restimulation and MGIA.

#### 2.5.2 Restimulation Assay

Splenocytes were restimulated using PPD‐T (purified protein derivative of *M. tb*). Two million cells were added to each well in a 96‐well round‐bottom plate (Thermo Scientific, Wilmington, DE, USA) in a volume of 200 μL and restimulated separately with 2 μg/mL of PPD‐T. After 2 h of incubation at 37°C in a CO_2_ incubator, a protein transport inhibitor Golgi plug (BD) was added to each well and further incubated for 4 h. The plate was removed and kept at 4°C overnight for flow staining of cells.

#### 2.5.3 Cytokine Staining

Restimulated splenocytes were washed with PBS containing 2% Bovine Serum Albumin (BSA) and stained with 10 μL live/dead aqua fixable dye (Invitrogen, Paisley, UK) for 10 min at room temperature in a V‐bottom 96 well plate (Thermo Scientific, Wilmington, DE, USA). An antibody mix for T cell surface markers was prepared using the following antibodies: CD3 BV605 (Clone 17A2, BioLegend, CA, USA), CD4 AF700 (Clone GK1.5, BioLegend, CA, USA), CD8a APC‐eF780 (clone 53–6.7, eBioscience, Hatfield, UK), CD62L PE‐Cy7 (MEL‐14, eBioscience, Hatfield, UK), CD44 BV711 (clone IM7, BioLegend, CA, USA) and CD127 E450 (clone A7R34, eBioscience, Hatfield, UK) with concentrations ranging from 2 to 4 μg/mL. After the live/dead staining, the cells were washed and stained with 40 μL per well of the surface marker antibody mixture containing Fc block (α‐FcγRIII) and incubated for 30 min at 4°C in the dark. The cells were washed and centrifuged at 1500 rpm in 200 μL 2% BSA/PBS (Sigma‐Aldrich, Dorset, UK) and fixed with 100 μL of the cell fixation and permeabilization solution cytofix/cytoperm (BD Bioscience, Oxford, UK) for 10 min at 4°C in the dark. A mixture of the following antibodies for T cell intracellular markers: IFN‐γ APC (clone XMG1.2, eBioscience, Hatfield, UK), IL‐17 PERCP‐Cy5.5 (clone TC11‐18H10.1, BioLegend, CA, USA), IL‐4 PE (clone 11B11, BioLegend, CA, USA), TNF‐α AF488 (clone MP6‐XT22, eBioscience, Hatfield, UK) was prepared in perm/wash. The cells were washed twice with perm/wash, and 40 μL per well of antibody mix was added and incubated for 30 min at 4°C. The stained cells were washed twice with perm/wash and twice with 2% BSA/PBS before resuspension for acquisition on a BD LSRII flow cytometer (Bioscience, NJ, USA).

#### 2.5.4 Mycobacterial Growth Inhibition Assay (MGIA)

A previously described protocol by Zelmer and colleagues was used with slight modifications [28]. Splenocytes were resuspended in a complete medium (10% FBS in RPMI‐1640 with HEPES modification and 2 mM L‐Glutamine) without antibiotics to achieve the required number of cells per ml for the assay. BCG Pasteur Aeras was used as a surrogate for *M. tb*. 5 million cells in a volume of 300 μL were added per well to a 48 well cell culture plate (Thermo Scientific, Wilmington, DE, USA). Mycobacteria were serially diluted in a sufficient volume of complete medium for all samples to a concentration of 100 CFU per 300 μL, and 300 μL per well was added to the splenocytes. The co‐culture was then incubated at 37°C in a CO_2_ incubator for 96 h. The co‐culture was then transferred into 2 mL screw cap tubes and centrifuged at 12000 rpm in a bench‐top Eppendorf microcentrifuge. The supernatants were removed leaving 100 μL each, and 400 μL of sterile tissue culture grade water was added to each well for 10 min to lyse the cells before addition to its corresponding pellet in the 2 mL tube. The tubes were then vortexed briefly. MGIT PANTA (Polymyxin B, Amphotericin B, Nalidixic Acid, Trimethoprim, Azlocillin) enrichment was reconstituted by adding lyophilized MGIT PANTA to MGIT growth supplement. Eight hundred microlitres of MGIT PANTA enrichment was added to each BACTEC MGIT tube and each cell lysate was added to a MGIT tube and incubated in a BACTEC MGIT liquid culture system (BD) until positivity was recorded. Two MGIT tubes were inoculated by adding 500 μL of mycobacterial to each tube as direct‐to‐MGIT controls on day 0 to normalise data across experiments.

A stock standard curve was used to convert time to positivity (TTP) to mycobacterial numbers (CFU). This was achieved using linear regression analysis on GraphPad Prism version 6, and the resulting equation was used to convert TTP to CFU. The standard curve was generated by inoculating 500 μL of 10‐fold dilutions of the mycobacterial strains into the MGIT tubes, and TTP was plotted against CFU obtained from plating aliquots of the mycobacteria on 7H11 agar plates containing 10% OADC supplement and 0.5% glycerol.

### 2.6 Statistics

Statistical significance was determined using the student t‐test (comparison of two groups) or one‐way ANOVA followed by Tukey's test for multiple comparisons (comparison between multiple groups) on GraphPad Prism version 6, while the Mann–Whitney test was used to determine statistical significance for cytokine secretion. FlowJo version 10 and GraphPad Prism version 6 were used for analysis. A value of *p* < 0.05 was considered statistically significant.

### 2.7 Study Approval

Animal studies were conducted in the Oxford University Biomedical Sciences Building which has full Home Office regulatory approval. Mice were used in accordance with the UK Animals (Scientific Procedures) Act under project licence number P9804B4F1 granted by the UK Home Office following ethical review and endorsement by the University of Oxford Animal Welfare and Ethical Review Board (AWERB).

## 3 Results

### 3.1 Infection With *P. yoelii 17XNL* Subsequent to BCG Vaccination Does Not Impair Ex Vivo Control of Mycobacterial Growth as a Surrogate of Vaccine Efficacy

**FIGURE 1:**
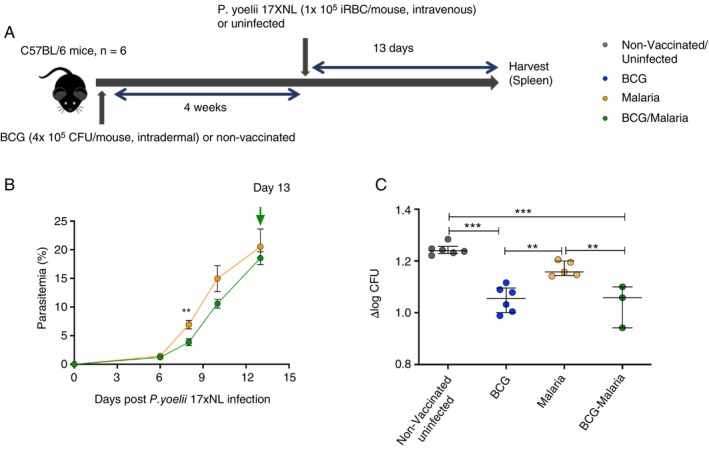
*P. yoelii 17XNL* infection post BCG vaccination does not reduce vaccine efficacy. Six C57BL/6 mice per group were injected intravenously with a non‐lethal species of *P. yoelii 17XNL* (*P. yoelii 17XNL*) 4 weeks after BCG vaccination. Thin blood smears were made from blood collected by tail vein prick every 2 or 3 days following the malaria infection and stained with Giemsa for parasite counts by light microscopy. The mice were killed 13 days after *P. yoelii 17XNL* infection and splenocytes restimulated with *P. yoelii 17XNL* peptide pool (PPt) for intracellular cytokine staining. (A) Study design. (B) Parasitaemia of *P. yoelii 17XNL* infected non‐vaccinated or BCG vaccinated mice over a 13‐day time course. Data are represented as the mean and standard error of the mean from six mice per group. Statistical significance between the two groups at specific time points was determined using the two‐tailed unpaired student *t*‐test. (C) Bacterial load quantified by MGIA. Data are presented as a fold change in bacterial burden (∆log CFU). Each data point represents an individual mouse, and the lines represent the median. Statistical significance was determined using the One‐way ANOVA with Turkey's multiple comparisons on GraphPad prism. *p ≤ 0.05, **p ≤ 0.01 and ***p ≤ 0.001.

Four groups of mice, namely, non‐vaccinated and uninfected controls, BCG vaccinated (BCG), BCG vaccinated followed by *P. yoelii 17XNL* infection 4 weeks later (BCG‐Malaria), and non‐vaccinated but *P. yoelii 17XNL* infected (Malaria) were included in the study; the study design is shown in the graphic (Figure 1A). Parasite growth was monitored by Giemsa‐stained thin blood smears until Day 13 when the mice were euthanised and spleens collected to perform the MGIA. Parasites were detected in blood at Day 3 after infection; a delayed increase in parasite burden was observed until Day 6, followed by an exponential increase of parasitaemia in both BCG vaccinated and non‐vaccinated mice. Interestingly, a transient increase in parasitaemia was observed on Day 8 in the BCG vaccinated group compared to the non‐vaccinated group. Nonetheless, there was no significant difference in parasitaemia levels between the BCG vaccinated and non‐vaccinated mice by Day 13 when the mice were euthanised. Parasitaemia levels reached approximately 20% in both groups (Figure 1B).

In the MGIA, as expected, a significant decrease in CFU (*p* < 0.0001, 0.2 log 10) was observed in the BCG vaccinated (median = 1.056) compared to non‐vaccinated mice (median = 1.240), but no differences (*p* = 0.0609) were detected between uninfected (median = 1.240) and *P. yoelii 17XNL* infected mice (median = 1.158) (Figure 1C). Of note, there was no significant difference in CFU between the BCG and BCG‐malaria groups, indicating BCG vaccinated mice were able to control mycobacterial growth to a similar extent with or without *P. yoelii 17XNL* infection.

### 3.2 *P. yoelii 17XNL* Infection Alters BCG Vaccine Induced PPD‐T Specific T Cell Cytokine Responses

**FIGURE 2:**
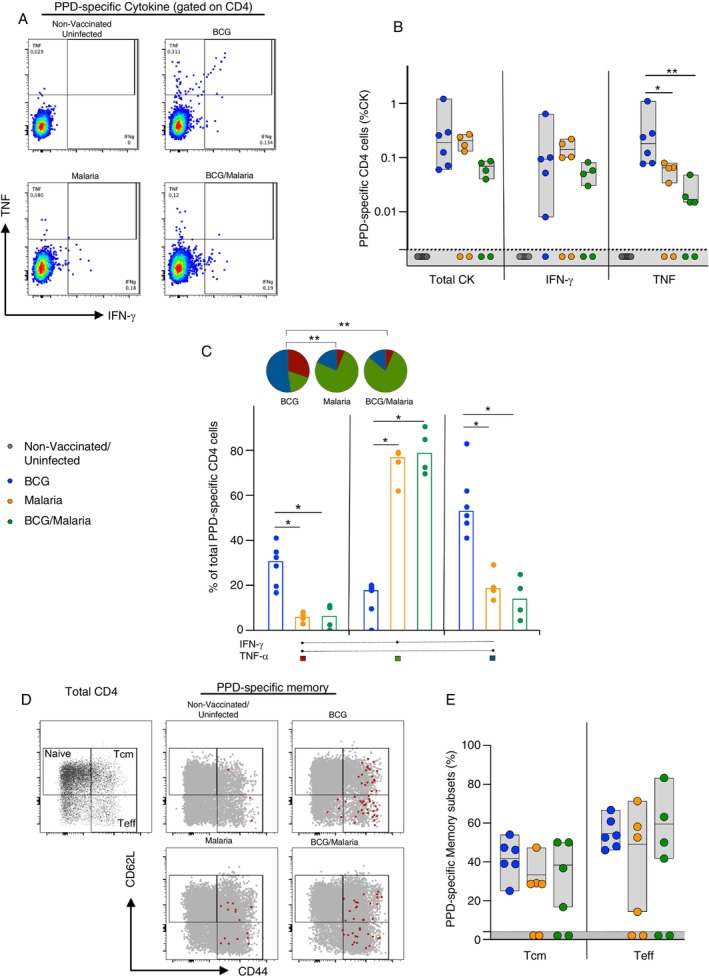
Magnitude of PPD‐specific Th1 CD4^+^ T cell cytokines post *P. yoelii 17XNL* infection of BCG vaccinated mice. C57BL/6 mice were infected with *P. yoelii 17XNL* 4 weeks after BCG vaccination. Four groups of 6 mice each were used for the experiment namely, naïve, BCG, BCG‐Malaria, and malaria The mice were killed 13 days after *P. yoelii 17XNL* infection and splenocytes restimulated with PPD‐T for intracellular cytokine staining. (A) Representative dot plots of PPD‐T‐specific T cells. (B) Percentage of total cytokine, IFNγ^+^ and TNF^+^ secreting CD4 T cells, respectively. Statistical significance was determined using the Mann–Whitney test on GraphPad Prism version (C) Percentage of multifunctional CD4^+^ cytokine secreting T cells in mice with PPD‐T‐specific responses. Each data point represents an individual animal, and the bars represent the median. Statistical significance was determined using the Wilkoxon Rank Sum test on Spice 6. (D) Representative plots showing Total PPD‐specific CD4^+^ Tcells overlayed onto memory phenotypes (E) PPD‐specific central memory and effector T cells. Statistical significance was determined using the Mann–Whitney test on GraphPad Prism version 6. *p ≤ 0.05, **p ≤ 0.01 and ***p ≤ 0.001.

To determine the effect of a malarial infection on T cell immunity following BCG vaccination, we compared the magnitude, functionality, and memory phenotype of CD4^+^ T cells 13 days after *P. yoelii 17XNL* infection (41 days after BCG vaccination). Splenocytes were isolated from the four groups (Uninfected, BCG, BCG‐Malaria, and Malaria) described above (Figure 1A), restimulated with PPD‐T, and intracellular IFN‐γ and TNF‐α expression was analysed by multiparameter flow cytometry. Figure 2A shows representative dot plots of IFN‐γ and TNF‐α producing CD4^+^ T cells following stimulation with PPD‐T. Data plotted in Figure 2B represent background subtracted values of total cytokine‐producing CD4^+^ T cells. Following stimulation with PPD‐T, there were no measurable cytokine producing CD4^+^ T cells in unvaccinated and uninfected mice, but robust responses were observed in splenocytes from the BCG vaccinated group (median total CK response: 0.19%). There was no significant reduction in the magnitude of either total cytokine (median: 0.19% vs. 0.069% for BCG and BCG‐malaria groups, respectively) or IFN‐γ (median: 0.094% vs. 0.054% for BCG and BCG‐malaria groups, respectively) responses in the BCG‐malaria group compared to the BCG only group. Interestingly, however, a significant decrease in TNF‐α (*p* = 0.0095) was detected in the BCG‐malaria group compared to the BCG group (median: 0.017% vs. 0.18%, respectively) (Figure 2B). Of note, PPD‐T stimulation also induced cytokine production in the malaria infected but unvaccinated control group, suggesting a degree of cross‐reactivity between *P. yoelii 17XNL*‐specific and PPD‐T‐specific T cells. Next, we assessed the functionality of the PPD‐T‐specific T cells. The overall functional profile of PPD‐T‐specific CD4^+^ T cells induced by BCG vaccination alone was significantly distinct from that in the malaria or BCG‐Malaria groups (*p* = 0.0016 and 0.0019, respectively, Figure 2C, pie charts). PPD‐T‐specific CD4^+^ T cells in the BCG group were characterised by a significantly higher frequency of IFN‐γ^+^ TNF‐α^+^ producing CD4^+^ T cells compared to the Malaria (*p* = 0.011) and BCG‐Malaria (*p* = 0.011) groups, as well as an increased frequency of IFN‐γ^−^ TNF‐α^+^ CD4^+^ T cells compared to the Malaria (*p* = 0.011) and BCG‐Malaria (*p* = 0.011) groups. In contrast, the BCG‐Malaria and malaria groups had a significantly larger proportion of IFN‐γ^+^ TNF‐α^−^ CD4^+^ T cells (*p* = 0.011 and *p* = 0.011) (Figure 2C, bar charts). No differences were detected between the BCG‐Malaria and malaria groups.

To investigate the phenotypic characteristics of PPD‐T‐specific T cell responses, we analysed memory cell populations (Figure 2D,E). The measurement of CD44 and CD62L expression enabled the detection of three distinct CD4^+^ T cell populations, namely naïve (CD44^−^CD62L^+^), central memory (Tcm: CD44^+^CD62L^+^), and effector (Teff: CD44^+^CD62L^−^) populations (Figure 2D). The memory profile of PPD‐T‐specific CD4^+^ T cells after BCG vaccination displayed no significant differences in Tcm (median: 42.65% vs. 28.8% vs. 43.4% for BCG, Malaria and BCG‐Malaria groups, respectively) or Teff subsets in any of the groups assessed (median: 53.15% vs. 55.35% vs. 56.6%, respectively; Figure 2E).

Although *P. yoelii 17XNL* infection has no significant effect on memory T cell populations following BCG vaccination, the overall data show that *P. yoelii 17XNL* infection alters BCG vaccine induced PPD‐T specific T cell cytokine responses.

### 3.3 Concurrent *P. yoelii 17XNL* Infection at the Time of BCG Vaccination Limits Ex Vivo Control of Mycobacterial Growth as a Surrogate of Vaccine Efficacy

We next investigated the effect of malaria infection prior to BCG vaccination on the PPD‐T‐specific cytokine responses and assessed mycobacterial growth inhibition as a surrogate of vaccine efficacy.

**FIGURE 3:**
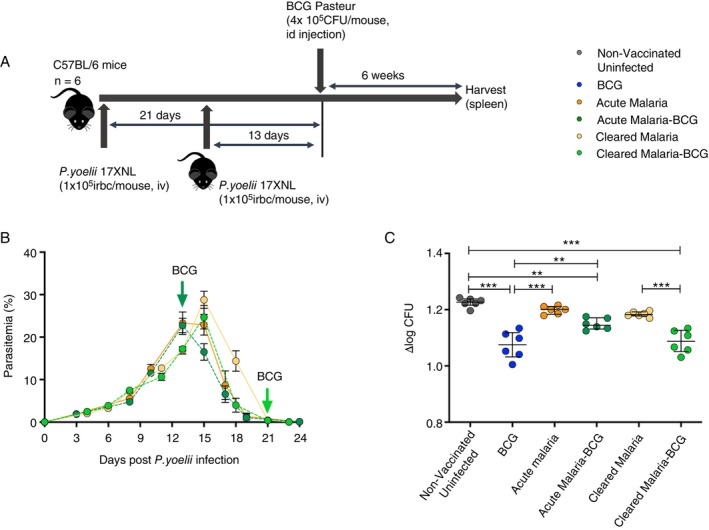
An acute malaria infection prior to BCG vaccination decreases the efficacy of BCG vaccination. An ex vivo mycobacterial growth inhibition assay was performed following BCG vaccination of C57BL/6 mice (six mice per group) at 13 days (during an active malaria infection) and 21 days (during a cleared malaria infection). Thin blood smears were made from blood collected by tail vein prick every 2 or 3 days following the malaria infection and stained with Giemsa for parasite counts by light microscopy. The mice were killed 6 weeks after BCG vaccination and 3 × 10^6^ splenocytes from each mouse were co‐cultured with 100 CFU of BCG Pasteur Aeras for 96 h. The cells were lysed and transferred to the BACTEC Mycobacterial growth inhibition tubes which were incubated in the BD BACTEC MGIT instrument until positivity was reached. (A) Timeline for malaria infection and BCG vaccination. (B) Parasitemia levels during *P. yoelii 17XNL* infection showing the time points at which BCG vaccination was done. (C) Bacterial load quantified by MGIA. MGIA data are presented as a fold change in bacterial burden ∆log CFU. Each data point represents an individual mouse, and the lines represent the median. Statistical significance was determined using the One‐way ANOVA with Turkey's multiple comparisons on GraphPad prism. *p ≤ 0.05, **p ≤ 0.01 and ***p ≤ 0.001.

Six groups of mice were used for the study, namely: unvaccinated and uninfected controls, BCG vaccinated (BCG), BCG vaccinated during 13 days after malaria infection (Acute Malaria‐BCG), unvaccinated but with acute malaria infection (Acute Malaria), BCG vaccinated 21 days after malaria infection (Cleared Malaria‐BCG), and unvaccinated with cleared malaria (Cleared Malaria) (Figure 3A). Parasitaemia levels were monitored by Giemsa‐stained thin blood smears made from blood collected by tail vein prick every 2 or 3 days after malaria infection.

The mice were euthanised 41 days after BCG vaccination and the expected BCG vaccine effect was observed using MGIA. Figure 3A shows the timeline for the malaria infections and BCG vaccination for the different experimental and control groups.

A slow increase in parasitaemia was observed until day 6, followed by the rapid multiplication of parasites in all four malaria groups (Figure 3B). The group that received BCG vaccination during acute malaria (Acute Malaria‐BCG) and the corresponding unvaccinated control (Acute Malaria) peaked around day 13, while the group that received BCG when malaria had cleared (Cleared Malaria‐BCG) and the corresponding unvaccinated control (Cleared Malaria) peaked a day later. The peak parasitaemia was approximately 25%–28% in all four groups, and there were no significant differences in parasitaemia levels between the experimental and control groups. Resolution of parasitaemia occurred in all four groups by day 21.

An enhancement in control of mycobacterial growth (approximately 0.2 log; *p* < 0.0001) was observed in the BCG vaccinated and uninfected group (median: 1.075) compared to the unvaccinated and uninfected control (median: 1.227) (Figure 3C). No significant differences in control of mycobacterial growth were detected between unvaccinated and uninfected (median: 1.227), acute malaria (median: 1.201) or cleared malarial control groups (median: 1.183). Mice that were vaccinated during acute malaria (acute malaria‐BCG group) displayed intermediate bacilli burdens (median: 1.144) which were significantly less than the unvaccinated uninfected control group (median: 1.227, *p* = 0.001) but significantly higher than the uninfected BCG vaccinated group (median: 1.075, *p* = 0.0014) and, more notably, inhibition of mycobacterial growth in this group was similar to the unvaccinated acute malaria control group (median: 1.201). Collectively, the data suggest that acute malaria at the time of BCG vaccination impairs the ability of BCG vaccinated mice to control the growth of mycobacteria.

In contrast, no differences in mycobacterial growth control were detected between the group that received BCG when malaria had cleared (cleared malaria‐BCG, median: 1.088) and the uninfected BCG vaccinated group (median: 1.075). This, together with the finding that a significant decrease in log_10_ CFU (approximately 0.2 log) was detected between the Cleared Malaria‐BCG group (median: 1.088) compared to the unvaccinated uninfected (median: 1.227, *p* < 0.0001) or Cleared Malaria unvaccinated control groups (median: 1183, *p* < 0.0001) suggests that the Cleared Malaria‐BCG group was able to control mycobacterial growth to a similar extent as the BCG vaccinated uninfected group. Therefore, an acute malaria infection, but not a recently cleared malarial infection, at the time of BCG vaccination may impair vaccine efficacy, as demonstrated by increased bacilli burdens in an ex vivo mycobacterial growth inhibition assay.

### 3.4 Acute *P. yoelii 17XNL* Infection Prior to BCG Vaccination Dampens Th1 Immunity

We also investigated the impact of malaria exposure either prior to, or at the time of BCG vaccination on the immunogenicity of the BCG vaccine. Splenocytes from the six groups of mice described previously were analysed for expression of the cytokines IFN‐γ or TNF‐α following stimulation with PPD‐T.

**FIGURE 4:**
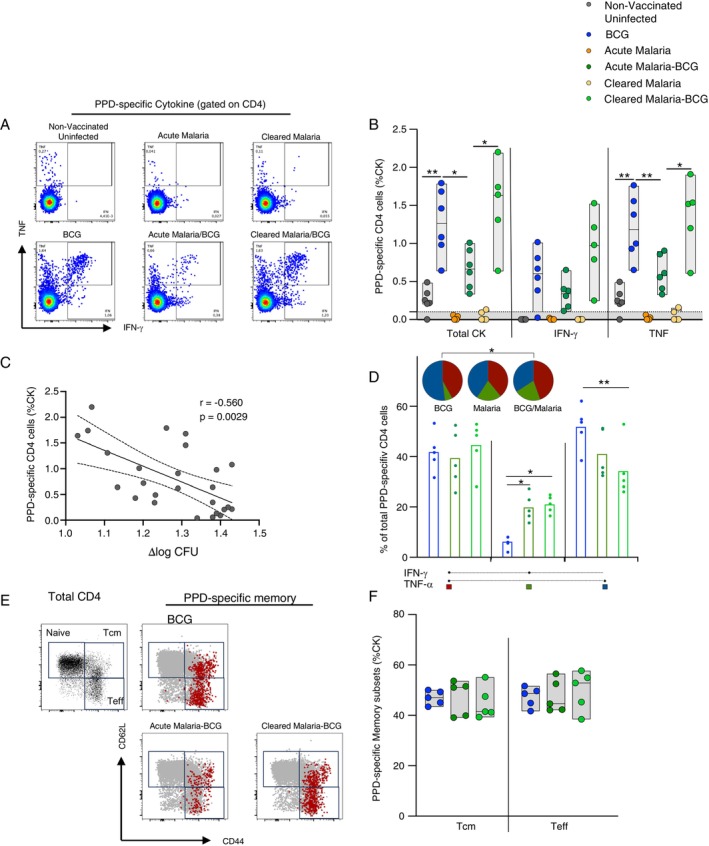
Acute malaria infection prior to BCG vaccination decreases Th1 cytokine responses. C57BL/6 mice (5–6 mice per group) were vaccinated with BCG Pasteur at 13 days (during an acute malaria infection) or 21 days (during a cleared malaria infection). The mice were killed 6 weeks after BCG vaccination and splenocytes restimulated with PPD‐T for intracellular cytokine staining. (A) Representative dot plots of PPD‐T‐specific T cells. (B) Percentage of total cytokine, IFNγ^+^ and TNF^+^ secreting CD4 T cells, respectively. Statistical significance was determined using the Mann–Whitney test on GraphPad Prism version (C) The total cytokine response comprising any CD4 T cell producing IFNγ^+^ and/or TNF^+^ was correlated to the *M. tb* bacterial load quantified by MGIA. Each data point represents an individual mouse, and the lines represent the median. Statistical significance was determined using a Spearman correlation analysis on GraphPad prism. (D) Proportion of multifunctional CD4^+^ cytokine secreting T cells. Each data point represents an individual animal, and the bars represent the median. Statistical significance was determined using the Wilkoxon Rank Sum test on Spice 6. (E) Representative plots showing Total PPD‐specific CD4^+^ T cells overlayed onto memory phenotypes (F) PPD‐specific central memory and effector T cells. Statistical significance was determined using the Mann–Whitney test on GraphPad Prism version 6. *p ≤ 0.05, **p ≤ 0.01 and ***p ≤ 0.001.

Figure 4A shows representative dot plots of IFN‐γ and TNF‐α producing CD4^+^ T cells following stimulation with PPD‐T. Stimulation with PPD‐T resulted in limited cytokine producing CD4^+^ T cells in splenocytes from unvaccinated uninfected mice (median total CK response: 0.25%) but robust responses in splenocytes from BCG vaccinated mice (median total CK response: 1.27%; *p* = 0.0043). Notably, PPD‐T stimulation induced no cytokine production in the acute malaria and only negligible production in the cleared malaria control groups. Comparison of the magnitude of the PPD‐T‐specific CD4^+^ T cell responses between the different BCG vaccinated groups, identified a significantly lower total cytokine and TNF‐α response in Acute Malaria‐BCG compared to either the BCG vaccinated uninfected (*p* = 0.026 and *p* = 0.0087 for total cytokine and TNF‐α, respectively) or Cleared Malaria‐BCG (*p* = 0.03 and *p* = 0.022 for total cytokine and TNF‐α, respectively) groups (median: 0.67% vs. 1.27% and 1.64% for total cytokine; median: 0.58% vs. 1.19% and 1.52% for TNF), with the BCG and Cleared Malaria‐BCG groups producing a similar magnitude of responses. Although no significant differences were detected in the magnitude IFN‐γ response between groups, a similar trend of a lower magnitude in the Acute Malaria‐BCG group was noted (median: 0.61% vs. 0.34% vs. 0.98 for BCG, Acute Malaria‐BCG and Cleared Malaria‐BCG, respectively) (Figures 4B and S1). Of note, the differences in the magnitude of the cytokine response between groups mirror that observed for mycobacterial growth control determined by MGIA, and there is a strong negative correlation between the total cytokine response and mycobacterial growth (*p* = 0.0029, Figure 4C). This correlation was generated by matching the CFU load (Figure S1) and total cytokine values (Figure 4B) from the splenocytes of the same mouse.

We next performed functional (Figure 4D) and phenotypic analysis (Figure 4E,F) on splenocytes from the BCG, Acute Malaria‐BCG, and Cleared Malaria‐BCG groups in which we had detected both IFN‐γ^+^ and TNF‐α^+^ CD4^+^ T cells. The overall functional profile of PPD‐T‐specific CD4^+^ T cells induced by BCG vaccination was distinct from that of the Cleared Malaria‐BCG group (*p* = 0.029; Figure 4D, pie charts); however, no significant differences in the proportion of dual functional IFN‐γ and TNF‐α producing CD4^+^ T cells were detected between the BCG, Acute Malaria‐BCG or Cleared Malaria‐BCG groups. Interestingly, a significant decrease in the frequency of IFN‐γ^+^ TNF‐α^−^ CD4^+^ T cells producing IFN‐γ alone was detected between the BCG group and both the Acute Malaria‐BCG and Cleared Malaria‐BCG groups (*p* = 0.028 and *p* = 0.009, respectively), while IFN‐γ^−^ TNF‐α^+^ producing CD4^+^ T cells were significantly lower in the BCG vaccinated group compared to the Cleared Malaria‐BCG group (*p* = 0.009). No differences were detected between the Acute Malaria‐BCG and Cleared Malaria‐BCG groups (Figure 4D).

To investigate the effect of acute malaria infection on the phenotypes of PPD‐T‐specific memory T cells, we analysed naïve (CD44−CD62L+), central memory (Tcm: CD44+CD62L+), and effector (Teff: CD44+CD62L−) populations (Figure 4E).

Phenotypic analysis of the PPD‐T‐specific CD4^+^ T cells displayed no significant differences in Tcm (median: 47% vs. 51% vs. 41.5% for BCG, Acute Malaria‐BCG and Cleared Malaria‐BCG‐Malaria, respectively) or Teff subsets in any of the 3 groups assessed (median: 48.6% vs. 44.6% vs. 52.8% for BCG, Acute Malaria‐BCG and Cleared Malaria‐BCG‐Malaria, respectively; Figure 4F). Therefore, the occurrence of acute malaria infection during BCG vaccination dampens the BCG‐specific Th1 immune responses, which may influence protective efficacy against TB.

## 4 Discussion

An improved understanding of the impact of malaria infection on BCG vaccine responses, particularly in areas where both malaria and TB are prevalent, is crucial for the development and testing of novel TB vaccine candidates as well as to inform vaccination policy in malaria‐endemic areas. Variation in BCG vaccine efficacy in the control of TB has been attributed in part to infections with parasites and other unrelated pathogens [33, 34]. More so, the timing of BCG vaccination with regard to malaria exposure may be crucial in determining the outcome of vaccine efficacy. While delaying BCG vaccination from birth to 10 weeks of age may enhance the quantitative and qualitative BCG‐specific T cell response [25], a delay may increase the likelihood that the infant is exposed to malaria infection before BCG administration. In such a scenario, the impact of prior exposure to malaria on the efficacy of subsequent BCG vaccination is uncertain. This is of particular concern in the post‐COVID‐19 pandemic era where ‘catch‐up’ vaccination programmes have been introduced to mitigate increased TB incidence due to low vaccine coverage during the pandemic. Therefore, an in‐depth understanding of the impact of the timing of malaria infection on BCG vaccination is crucial.

In this study, we postulated that the timing of BCG vaccination with respect to malaria infection can influence BCG‐induced immune responses and control of *M. tb*. We investigated the effect of malaria infection on the PPD‐T‐specific T cell responses induced by BCG vaccination and the outcome on the control of mycobacterial growth ex vivo by MGIA.

We have previously demonstrated that neither virulent *P. berghei* nor low virulence *P. chabaudi* infection post‐BCG vaccination results in vaccine failure [26]. In this study, we made use of a *P. yoelii 17XNL* mouse infection model which has been widely used in other studies to draw correlates with human malaria [35]. These rodent malarial parasites show a high level of gene orthology with human malaria parasites, supporting their use in experimental models [36]. C57BL/6 mouse strains are resistant to *M. tb* challenge and able to control lung infection, and previous studies have successfully used C57BL/6 mice in a coinfection model of TB and malaria [37]. Similar to our previous reports, we found that *P. yoelii 17XNL* infection after BCG vaccination did not abrogate control of mycobacterial growth ex vivo by MGIA. This is also concordant with findings by Parra et al. [32] showing retained BCG vaccine efficacy despite *P. yoelii 17XNL* infection.

We investigated the impact of *P. yoelii 17XNL* infection prior to BCG vaccination on the outcome of control of mycobacterial growth ex vivo by MGIA. We report that prior exposure to malaria infection does not hamper the protective efficacy of BCG vaccination ex vivo. A significant decrease in bacilli burdens in the groups that received BCG after cleared malaria compared to all non‐vaccinated controls was observed, suggesting that splenocytes from these mice were able to control mycobacterial growth in culture. Surprisingly, the group of mice that received BCG vaccination during an acute malaria infection was not able to control mycobacterial growth to the same extent as the BCG vaccinated uninfected group and the Cleared Malaria–BCG group. BCG vaccination resulted in a 0.2 log reduction in CFU in the splenocyte MGIA. It is worth noting that in our study we could not distinguish between the vaccine BCG Pasteur and the BCG Aeras in the MGIA using BACTEC MGIT quantification. Hence, the presence of residual BCG in splenocytes from vaccinated mice might result in an underestimation of growth inhibition in these groups in the MGIA [38].

BCG and malaria both induce a typical Th1 immune response characterised by the secretion of the cytokine IFN‐γ by CD4^+^ T cells [39, 40, 41]. A robust Th1 immune response including TNF‐α and IFN‐γ is critical to the control of *M. tb* infection [42]. Individuals with defects in IFN‐γ signalling caused by a mutation in the IFN‐γ receptor gene are more susceptible to TB, and IFN‐γ deficiency has been associated with disseminated *M. tb* infection, highlighting the role of IFN‐γ in the control of *M. tb* [43]. Despite this, studies have shown that IFN‐γ is not a good correlate of protection since an excess can also lead to inflammatory damage and exacerbated disease [44]. The protective role of IFN‐γ in malaria has also been demonstrated in both humans and rodent malaria models [45]. IFN‐γ deficient mice infected with *P. yoelii 17XNL* or *P. chabaudi* succumb due to higher and prolonged blood parasitaemia compared to wild‐type mice [46]. We analysed IFN‐γ and TNF cytokine responses in mice that had *P. yoelii 17XNL* infection either post‐vaccination, prior to BCG vaccination, or during vaccination in the groups previously described above.

Mice infected with *P. yoelii 17XNL* post‐BCG vaccination showed no alterations in the frequency of total cytokine‐producing CD4^+^ T cells but a significant decrease in IFN‐γ/TNF‐α‐producing CD4^+^ T cells and TNF‐producing CD4^+^ T cells not co‐producing IFN‐γ compared to BCG vaccination alone. Our data is concordant with reports from Parra et al. showing reduced polyfunctional T cells during acute *P. yoelii 17XNL* infection. Interestingly, however, they demonstrated a recovery of the polyfunctional T cells at 7 and 10 weeks after clearance of *P. yoelii 17XNL* parasitaemia [32]. In both studies, this reduction in polyfunctional/bifunctional T cells did not translate in a reduction of BCG vaccine efficacy.

In contrast, we observed a significant decrease in the frequency of BCG‐specific total cytokine producing and TNF‐α producing CD4^+^ T cells in the group that was vaccinated during an acute *P. yoelii 17XNL* infection but not in the group that received the BCG vaccine when malaria had cleared. It should be noted that the parasitaemia had cleared in all the groups that had malaria prior to euthanasia. Interestingly, however, no differences were seen in IFN‐γ/TNF‐producing CD4^+^ T cells, although the malarial infection both during acute or convalescent *P. yoelii 17XNL* infection was associated with a higher frequency of IFN‐γ^+^ TNF‐α^−^ and a lower frequency of IFN‐γ^−^ TNF‐α^+^ CD4^+^ T cells.

In our studies, the frequency of total cytokine‐producing CD4^+^ T cells rather than functionality appears to correlate with ex vivo control of mycobacterial growth. This highlights a potential disconnect between bifunctional and IFN‐γ‐producing T cells and protective efficacy. This lack of strong correlates of protection against *M. tb* hampers early vaccine immunogenicity studies and thus novel vaccine development [47].

Studies have also suggested that antigen‐specific Tcm, as opposed to Teff cells, are required for optimal long‐term protection from *M. tuberculosis* [48, 49].

In our study however, no alterations in BCG‐specific memory T cell profiles were detected in mice that had *P. yoelii 17XNL* infection either post‐vaccination, prior to BCG vaccination, or during vaccination, suggesting the malarial infection may not lead to impairment of long‐term BCG vaccine protection.

Overall, the timing of BCG vaccination in relation to malaria infection may impact the protective efficacy of the BCG vaccine to *M. tb* with concomitant modulations in the T cell immune responses. This potentially has clinical implications where BCG vaccination is delayed in malaria‐endemic areas, but the effects could be mitigated by testing and treating children for sub‐clinical malarial infection prior to vaccination.

## Author Contributions

A.J.S. and H.M.c.S. designed the study. E.T., R.T. and R.K. analysed the data. A.J.S. acquired and managed ethical approval for the study. A.J.S. and N.P. performed animal experiments. E.T., N.P. and R.T. performed ex vivo experiments. E.T., R.K., N.J.H. and M.J. wrote the manuscript. All authors reviewed and approved the manuscript prior to submission.

## Disclosure

The authors have nothing to report.

## Conflicts of Interest

The authors declare no conflicts of interest.

## Supporting information


Data S1.

## Data Availability

The data that support the findings of this study are available from the corresponding author upon reasonable request.
