# Supplementary material for: Acute Plasmodium yoelii 17XNL Infection During BCG Vaccination Limits T Cell Responses and Mycobacterial Growth Inhibition
Source: Immunology. 2025 Jun 19;176(3):373–84. doi: 10.1111/imm.70006 (PMC12500385; doi:10.1111/imm.70006)
Supplement: Supplementary file 1 — Data S1. [file IMM-176-373-s001.docx]

**Supplementary Table 1:** Mouse distribution for study 1

| **Group** | **n** | **Analysis** | **Time-point (days)** | **Main Figure** | **Missing data points** | **Justification** |
| --- | --- | --- | --- | --- | --- | --- |
| Non-Vaccinated / Uninfected | 6 | Parasitaemia | 0-13 | 1B | N/A |  |
|  |  | Bacterial load | 41 | 1C | N/A |  |
|  |  | T cell analysis | 41 | 2A-E | N/A |  |
| BCG | 6 | Parasitaemia | 0-13 | 1B | N/A |  |
|  |  | Bacterial load | 41 | 1C | N/A |  |
|  |  | T cell analysis | 41 | 2A-E | N/A |  |
| Malaria | 6 | Parasitaemia | 0-13 | 1B | N/A | Contamination of MGIA culture |
|  |  | Bacterial load | 41 | 1C | 1 |  |
|  |  | T cell analysis | 41 | 2A-E | N/A |  |
| BCG/Malaria | 6 | Parasitaemia | 0-13 | 1B | N/A | Contamination of MGIA culture |
|  |  | Bacterial load | 41 | 1C | 3 |  |
|  |  | T cell analysis | 41 | 2A-E | N/A |  |

**Supplementary Table 2:** Mouse distribution for study 2

| **Group** | **n** | **Analysis** | **Time-point (days)** | **Main Figure** | **Supplementary Figure** | **Missing data points** |
| --- | --- | --- | --- | --- | --- | --- |
| Non-Vaccinated / Uninfected | 6 | Parasitaemia | 0-24 | 3B |  | N/A |
|  |  | Bacterial load | 42 (post BCG) | 3C |  | N/A |
|  |  | T cell analysis | 42 (post BCG) |  | 1 | N/A |
| BCG | 6 | Parasitaemia | 0-24 | 3B |  | N/A |
|  |  | Bacterial load | 42 (post BCG) | 3C |  | N/A |
|  |  | T cell analysis | 42 (post BCG) |  | 1 | N/A |
| Acute Malaria | 6 | Parasitaemia | 0-24 | 3B |  | N/A |
|  |  | Bacterial load | 42 (post BCG) | 3C |  | N/A |
|  |  | T cell analysis | 42 (post BCG) |  | 1 | N/A |
| Acute Malaria / BCG | 6 | Parasitaemia | 0-24 | 3B |  | N/A |
|  |  | Bacterial load | 42 (post BCG) | 3C |  | N/A |
|  |  | T cell analysis | 42 (post BCG) |  | 1 | N/A |
| Cleared Malaria | 6 | Parasitaemia | 0-24 | 3B |  | N/A |
|  |  | Bacterial load | 42 (post BCG) | 3C |  | N/A |
|  |  | T cell analysis | 42 (post BCG) |  | 1 | N/A |
| Cleared Malaria / BCG | 6 | Parasitaemia | 0-24 | 3B |  | N/A |
|  |  | Bacterial load | 42 (post BCG) | 3C |  | N/A |
|  |  | T cell analysis | 42 (post BCG) |  | 1 | N/A |

**Supplementary Table 3:** Mouse distribution for study 3

| **Group** | **n** | **Analysis** | **Time-point (days)** | **Main Figure** | **Missing data points** |
| --- | --- | --- | --- | --- | --- |
| Non-Vaccinated / Uninfected | 6 | Bacterial load | 42 (post BCG) | 4C | N/A |
|  |  | T cell analysis | 42 (post BCG) | 4A, B, D-F | N/A |
| BCG | 6 | Bacterial load | 42 (post BCG) | 4C | N/A |
|  |  | T cell analysis | 42 (post BCG) | 4A, B, D-F | N/A |
| Acute Malaria | 5 | Bacterial load | 42 (post BCG) | 4C | N/A |
|  |  | T cell analysis | 42 (post BCG) | 4A, B, D-F | N/A |
| Acute Malaria / BCG | 5 | Bacterial load | 42 (post BCG) | 4C | N/A |
|  |  | T cell analysis | 42 (post BCG) | 4A, B, D-F | N/A |
| Cleared Malaria | 5 | Bacterial load | 42 (post BCG) | 4C | N/A |
|  |  | T cell analysis | 42 (post BCG) | 4A, B, D-F | N/A |
| Cleared Malaria / BCG | 6 | Bacterial load | 42 (post BCG) | 4C | N/A |
|  |  | T cell analysis | 42 (post BCG) | 4A, B, D-F | N/A |


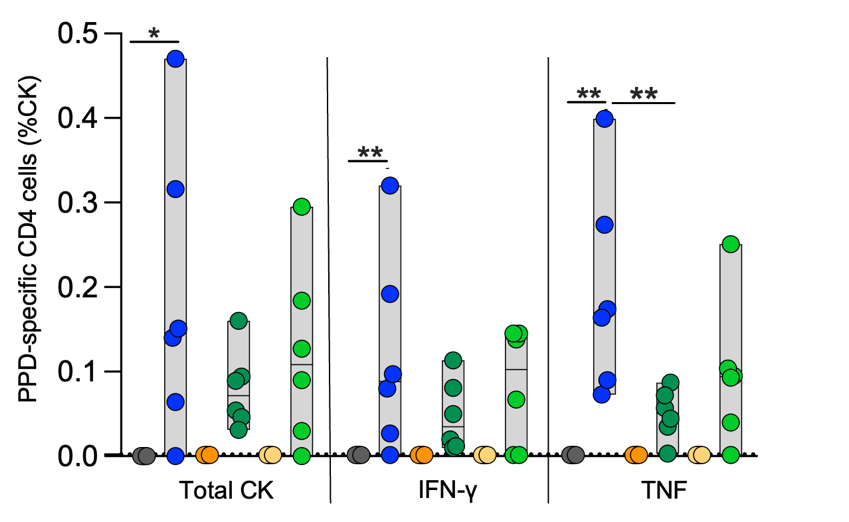


**Supplementary Figure 1**: Acute malaria infection prior to BCG vaccination decreases TNF cytokine responses. C57BL/6 mice (5-6 mice per group) were vaccinated with BCG Pasteur at 13 days (during an acute malaria infection) or 21 days (during a cleared malaria infection). The mice were killed six weeks after BCG vaccination and splenocytes restimulated with PPD-T for intracellular cytokine staining. A) Percentage of total cytokine, IFNγ^+^ and TNF^+^ secreting CD4 T cells respectively. Statistical significance was determined using the Mann-Whitney test on GraphPad Prism version *p≤0.05; **p≤0.01.
